# Ultrafast Spin Dynamics of Pt/Gd_19_(Co_0.8_Fe_0.2_)_81_/Ta Heterostructure Investigated by Double-Pump Terahertz Emission Spectroscopy

**DOI:** 10.3390/nano16070390

**Published:** 2026-03-24

**Authors:** Changwei Li, Bo Lu, Nuoxi Yu, Zhangshun Li, Haoran Xu, Huiping Zhang, Zuanming Jin

**Affiliations:** 1 Terahertz Technology Innovation Research Institute, Terahertz Spectrum and Imaging Technology Cooperative Innovation Center, Shanghai Key Laboratory of Modern Optical System, University of Shanghai for Science and Technology, Shanghai 200093, China; 2335051115@st.usst.edu.cn (C.L.); 233350665@st.usst.edu.cn (B.L.); 2335060308@st.usst.edu.cn (N.Y.); 231200051@st.usst.edu.cn (Z.L.); 223330700@st.usst.edu.cn (H.X.); 2 Shanghai Institute of Intelligent Science and Technology, Tongji University, Shanghai 200092, China

**Keywords:** optical pump–THz emission, ultrafast spin dynamics, ferrimagnetic heterostructure, spin–lattice interaction

## Abstract

Ultrafast spin dynamics is a core research focus for advancing ultrafast spintronic devices, yet its accurate quantitative probing remains a challenge with conventional time-resolved techniques. Herein, we employ double-pump optical pump–terahertz emission spectroscopy (OPTE) to investigate the ultrafast spin dynamics of a Pt/Gd_19_(Co_0.8_Fe_0.2_)_81_/Ta ferrimagnetic rare-earth–transition-metal heterostructure. Experimental measurements resolve a single-step ultrafast demagnetization process with a characteristic time of ~0.42 ± 0.02 ps, followed by two-stage magnetic recovery involving a fast relaxation and a slow relaxation process. The fast and slow recovery time constants show a distinct positive dependence on the control pump fluence, increasing from 2.49 ± 0.11 ps to 3.28 ± 0.03 ps and 57.36 ± 11.28 ps to 164.96 ± 1.61 ps, respectively, as the pump fluence rises from 0.80 to 1.19 mJ/cm^2^. The ~0.42 ps demagnetization timescale is consistent with that of 3d transition metals, indicating the transient magnetic response of the low-Gd-concentration heterostructure is dominated by the CoFe sublattice. Our findings validate that OPTE is an effective approach for the quantitative characterization of electron–lattice–spin coupling processes in spin-based heterostructures and provide critical experimental insights for controllable manipulation of ultrafast spin dynamics, laying a foundation for the design of ultrafast terahertz spintronic devices.

## 1. Introduction

Ultrafast demagnetization is the significant magnetization reduction in magnetic materials on sub-picosecond to several-picosecond timescales after femtosecond pulse excitation. First investigated in Ni thin films by Eric Beaurepaire et al. in 1996 [1], it has become a core topic in spintronics research [2,3,4,5,6]. Its ultrafast dynamics indicates an efficient angular momentum dissipation channel in ferromagnets [7,8]. By the Einstein–de Haas effect, spin angular momentum is ultimately transferred to the lattice [9,10]. The ultrashort laser–magnetic material interaction reveals nonequilibrium electron–lattice–spin coupling, and lays the experimental/theoretical foundation for ultrafast magnetic storage, terahertz (THz) spintronic devices, and optically controlled spin dynamics [11,12,13].

Regarding the underlying mechanisms, two primary scenarios explain laser-induced sub-picosecond demagnetization. The first is spin-flip scattering: nonequilibrium electrons excited by femtosecond pulses collide with phonons or impurities in the ferromagnet, triggering spin flips [14,15]. The second is super-diffusive spin transport [16,17]: the excited majority electrons have higher band velocities and longer lifetimes than the minority ones, depleting majority carriers and reducing magnetization in the excited region. Femtosecond laser-induced spin transport generates sub-picosecond spin-polarized currents, which convert to in-plane charge currents at ferromagnetic–nonmagnetic interfaces via spin–orbit coupling, ultimately producing THz radiation [18,19,20].

Experimentally, ultrafast demagnetization dynamics is characterized by various time-resolved techniques, such as the time-resolved magneto-optical Kerr effect (TR-MOKE) [21,22,23], time-resolved X-ray magnetic circular dichroism (TR-XMCD) [24,25], and optical second-harmonic generation (SHG) [26,27]. Though the TR-MOKE is a standard tool for studying ultrafast demagnetization and magnetization precession, quantifying its signals is non-trivial—its transient optical response does not directly reflect intrinsic magnetization evolution, but is modulated by the material’s wavelength-dependent optical and magneto-optical coefficients [28]. TR-XMCD and SHG are limited by radiation sources and optical penetration depth, respectively [29,30]. Thus, accurately probing ultrafast spin dynamics in materials and functional heterostructures under realistic device conditions remains a challenge.

With the rapid development of ultrafast THz spectroscopy, time-resolved optical pump–THz emission spectroscopy (OPTE) has emerged as a powerful platform for detecting and manipulating spin dynamics. Representative examples include recent studies on ultrafast spin dynamics in Fe thin films [31], antiferromagnetic–ferromagnetic phase transition in FeRh [32,33], and nonlinear magnetic responses in ferromagnetic heterostructures [34], demonstrating that OPTE offers a complementary and effective experimental approach for exploring ultrafast spin dynamics in ferromagnetic systems [35].

Pt/GdCoFe/Ta is a ferrimagnetic rare-earth and transition-metal heterostructure with great application potential. Its Gd–CoFe alloy layer has tunable ferromagnetism, and the antiparallel coupling between rare-earth Gd and transition metals Co/Fe endows it with rich magnon–lattice dynamical characteristics [36,37]. In this work, we use OPTE to study the ultrafast spin dynamics in a ferrimagnetic Pt/GdCoFe/Ta heterostructure. A single-step demagnetization process followed by two-stage magnetic recovery is observed. Our results provide insights into femtosecond-scale magnetic loss and recovery processes, and promote the advancement of ultrafast spintronics toward more controllable manipulation.

## 2. Materials and Methods

### 2.1. Sample Preparation

In this work, a Pt(3 nm)/Gd_19_(Co_0.8_Fe_0.2_)_81_(10 nm)/Ta(3 nm) heterostructure was deposited on the SiO_2_ substrate via a Kurt J. Lesker Company magnetron sputtering system (Jefferson Hills, PA, USA), with a base pressure of 3.0 × 10^−8^ torr. The 10 nm-thick Gd_19_(Co_0.8_Fe_0.2_)_81_ film was co-sputtered from Co_0.8_Fe_0.2_ and Gd targets, with precise Gd concentration control achieved by adjusting Gd target sputtering power while keeping the deposition time constant. A 3 nm-thick Ta buffer layer was first deposited on 0.5 mm-thick SiO_2_ substrates to enhance film adhesion and surface smoothness, followed by a 10 nm-thick Gd_19_(Co_0.8_Fe_0.2_)_81_ layer and a capping layer of Pt(3 nm-thick) to prevent oxidation. Due to the negative exchange coupling between Fe atoms’ 3d spin magnetic moment and Gd 4f orbitals’ magnetic moment, FeCo and Gd sublattices form a collinear antiferromagnetic coupling, such that the alloy film’s net magnetization is determined by Gd concentration.

### 2.2. Experimental Setup

**Figure 1:**
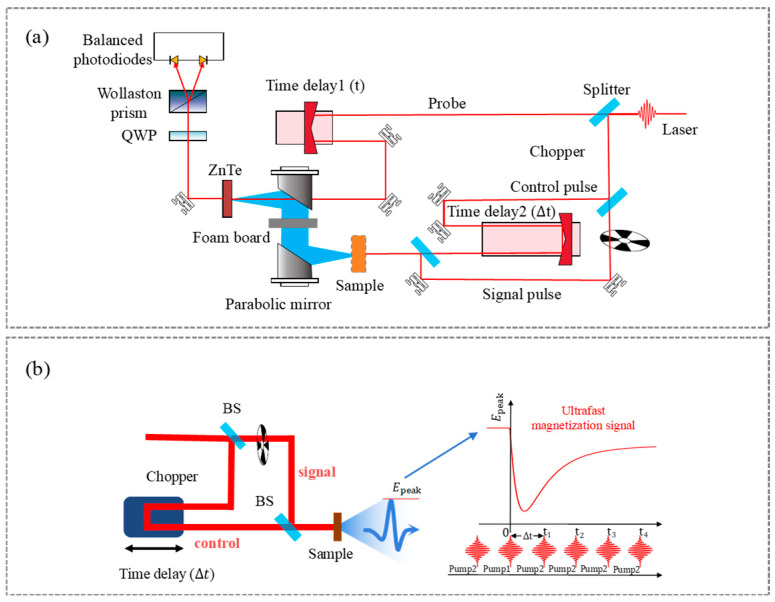
(**a**) Schematic of the double pump optical pump–THz emission (OPTE) optical setup. (**b**) Schematic illustration of the OPTE principle for probing ultrafast spin dynamics.

Figure 1a shows the configuration of the double-pump optical pump–THz emission (OPTE) setup. The system is driven by a 1 kHz repetition rate femtosecond laser (central wavelength: 800 nm and pulse duration: 35 fs) with linearly polarized output. The incident beam is split by a beam splitter into pump and probe branches: the pump beam excites the sample to emit THz radiation, which is collected by two off-axis parabolic mirrors into a (110)-oriented ZnTe electro-optic crystal (1 mm thick) for time-domain detection via electro-optic sampling. A precision delay stage (Time delay 1) in the probe beam path scans the pump–probe time delay t, enabling reconstruction of the complete THz temporal waveform. To realize a double-pump modulation scheme, the pump beam is further split by a second beam splitter into two independent excitation pulses: the signal pulse (Pump 1) and the control pulse (Pump 2). Pump 1 acts as the signal branch to generate THz radiation, while Pump 2 serves as the control pulse to induce transient modifications of the material’s magnetization state. The two pump beams are overlapped spatially and collinearly incident on the sample, with their relative timing precisely tuned via independent delay stages. A second delay line (Time delay 2) is introduced in the control-pulse beam’s path to adjust the temporal separation Δt relative to the signal pulse. An in-plane magnetic field of ±160 mT was applied to keep the sample in the saturated magnetization state, by an electromagnet.

In the OPTE experiment, the chopper is placed in the signal pump’s path, such that only the signal pulse is modulated according to the lock-in amplification principle. When the signal pump pulse passes through the chopper, the THz emission signal $E_{\mathrm{THz}}^{\mathrm{signal}\;\&\;\mathrm{control}}$ is generated by the combined action of the signal and control pump pulses. When the chopper blocks the signal pump pulse, only the unmodulated control pump pulse generates the THz emission signal $E_{\mathrm{THz}}^{\mathrm{control}}$. The measured signal is then obtained as the difference, $E_{\mathrm{THz}}^{\mathrm{signal}\;\&\;\mathrm{control}}-E_{\mathrm{THz}}^{\mathrm{control}}$. We can measure the change in the peak value of $E_{\mathrm{THz}}^{\mathrm{Signal}}$, $E_{\mathrm{peak}}\left(\Delta t\right)$, which is induced by ultrafast demagnetization using a control pump pulse.

Figure 1b illustrates the principle of OPTE for probing ultrafast spin dynamics. In the double-pump modulation measurement, Time delay 1 is fixed at the peak position of the emitted THz field (denoted as $E_{\mathrm{peak}}$), while Time delay 2 is scanned to adjust the temporal separation Δt between Pump 1 and Pump 2 pulses. The peak amplitude of the THz emission generated by the signal beam (Pump 1) $E_{\mathrm{peak}}$ is modulated by the sample’s transient magnetization change and subsequent recovery processes induced by the control beam (Pump 2). On this basis, the initial ultrafast demagnetization and the sub-picosecond to tens-of-picoseconds magnetic recovery dynamics can be experimentally resolved.

## 3. Results and Discussion

**Figure 2:**
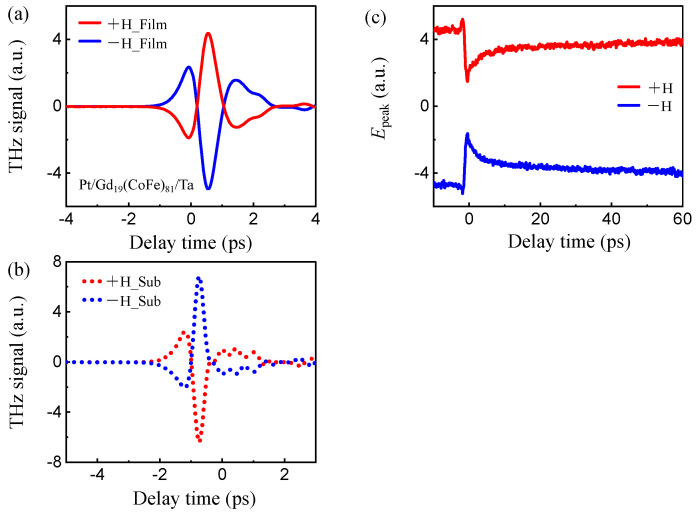
(**a**) THz time-domain signals measured from the Pt/Gd_19_(CoFe)_81_/Ta heterostructure with (**a**) film-side excitation and (**b**) substrate-side excitation under positive and negative magnetic fields. (**c**) Magnetic field-dependent, laser-induced modulation of the peak THz electric field $E_{\mathrm{peak}}$ as a function of the double-pump delay Δt under opposite magnetic fields. The pump fluences of signal- and control-pulse are 1.87 and 1.19 mJ/cm^2^, respectively.

Figure 2a presents the THz time-domain signals emitted from the film-side-pumped Pt/Gd_19_(CoFe)_81_/Ta/SiO_2_ heterostructure under opposite external magnetic fields (+H and −H). A clear polarity reversal of the THz electric field waveform is observed upon reversing the magnetic field direction, and the signal amplitude remains nearly unchanged. This symmetry indicates that the THz radiation originates from magnetization-related transient currents rather than from nonmagnetic effects such as thermal radiation or optical rectification. Figure 2b presents the THz time-domain signals measured under substrate-side excitation under identical experimental conditions. When the external magnetic field is reversed from +H to −H, the THz waveform exhibits a clear polarity reversal. Compared with the film-side excitation, applied with the same magnetic field direction, the substrate-side incidence reverses the polarity of the emitted THz radiation. Such behavior is consistent with previous reports on the inverse spin Hall effect (ISHE) in ferromagnet/heavy-metal multilayers [38,39]. The femtosecond optical laser induces ultrafast spin current $J_{S}$(t) in the ferromagnetic layer, which propagates toward the adjacent heavy-metal layer. Through the ISHE, $J_{S}$(t) is converted into a transverse charge current $J_{C}$(t), following $J_{C}\left(t\right)\propto J_{S}\left(t\right)\times \sigma$, where σ is the spin polarization, which is parallel to M. The time-dependent charge current $J_{C}\left(t\right)$ acts as a transient dipole source that radiates THz electromagnetic fields in the far field.

Figure 2c shows the OPTE signals $E_{\mathrm{peak}}\left(\Delta t\right)$ of the Pt/Gd_19_(CoFe)_81_/Ta heterostructure measured under positive and negative magnetic fields. Consistent with the emitted THz time-domain waveforms, the OPTE signal reverses its polarity upon magnetic field reversal. Note that the spin current generated by ultrafast demagnetization is proportional to M×dM/dt, even under magnetization reversal. However, the conversion of spin current into a charge current via the ISHE introduces an additional factor through the spin polarization σ. Therefore, the $J_{C}$ is odd with respect to magnetization reversal. This demonstrates that the OPTE signal reflects the evolution of magnetization, confirming the feasibility of this approach for investigating ultrafast spin dynamics. Three distinct dynamical processes can be identified: an initial sub-picosecond demagnetization characterized by a rapid signal decrease, followed by a fast magnetic recovery, and subsequently a slow relaxation process extending to nearly a hundred picoseconds.

**Figure 3:**
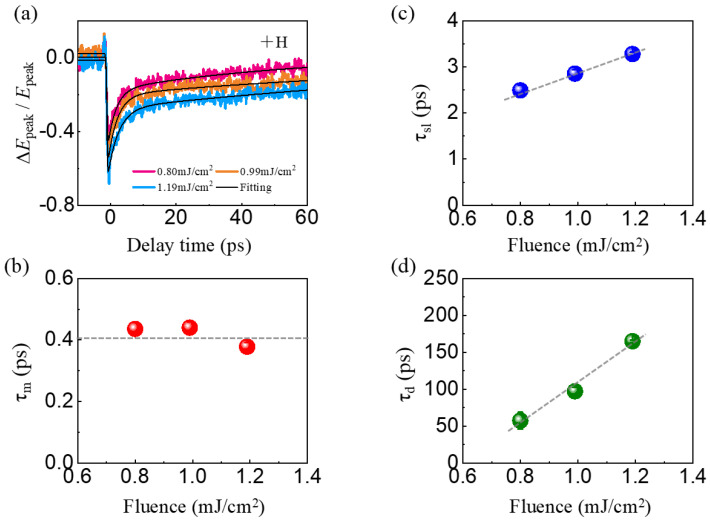
(**a**) OPTE signals $\frac{\Delta E_{\mathrm{peak}}\left(\Delta t\right)}{E_{\mathrm{pea}k}}$ for the Pt/Gd_19_(CoFe)_81_/Ta/SiO_2_ heterostructure under positive magnetic field measured with different control pulse fluence 0.80–1.19 mJ/cm^2^, while the signal fluence is fixed at 1.87 mJ/cm^2^. (**b**–**d**) Evolution of the ultrafast demagnetization time ($\tau_{m}$), fast recovery time ($\tau_{\mathrm{sl}}$), and slow recovery time ($\tau_{d}$) as functions of the control pulse fluence. The dashed lines represent the corresponding linear fitting results. The error bars are calculated based on the standard deviation extracted from the fitting procedure.

Figure 3a shows the control pulse fluence dependence of the normalized modulation signal $\Delta E_{\mathrm{peak}}\left(\Delta t\right)/E_{\mathrm{peak}}$ for the Pt/Gd_19_(CoFe)_81_/Ta heterostructure under positive magnetic fields, where $\Delta E_{\mathrm{peak}}\left(\Delta t\right)$ is obtained by subtracting the initial THz peak amplitude $E_{\mathrm{peak}}$ from $E_{\mathrm{peak}}\left(\Delta t\right)$. It can be found that the amplitude of $\Delta E_{\mathrm{peak}}\left(\Delta t\right)/E_{\mathrm{peak}}\propto \Delta M\left(\Delta t\right)$ increases monotonically with increasing pump fluence, rising from 50% at 0.8 mJ/cm^2^ to 68% at 1.19 mJ/cm^2^. This trend indicates that a stronger control pulse induces more pronounced demagnetization.

Analogous to conventional pump–probe spectroscopy, the temporal resolution of $\Delta E_{peak}\left(\Delta t\right)/E_{peak}$ is determined by the optical pulse duration rather than the THz pulse duration, which investigates spin dynamics. In order to quantitatively analyze the ultrafast spin dynamics observed in Figure 3a, it is required to obtain the time constants of the spin dynamics, experimental data were fitted with a phenomenological bi-exponential model [31]:

(1) $$\frac{\Delta E_{\mathrm{peak}}\left(\Delta t\right)}{E_{\mathrm{peak}}}=\left[1-\mathrm{erf}\left(-2\ln\left(2\right)\frac{\Delta t}{\tau_{m}}\right)\right]\times \left[\left(A-B\right)\exp\left(-\frac{\Delta t}{\tau_{\mathrm{sl}}}\right)+B\exp\left(-\frac{\Delta t}{\tau_{d}}\right)+C\right]$$

where parameters *A* and *B* show the demagnetization amplitudes before and after the fast recovery process, respectively. $\tau_{m}$ is on the sub-picosecond timescale and characterizes the ultrafast demagnetization process. The time constants $\tau_{\mathrm{sl}}$ and $\tau_{d}$ describe fast magnetic recovery and subsequent slow relaxation process, respectively. $\tau_{\mathrm{sl}}$ is associated with spin–lattice angular momentum transfer, while $\tau_{d}$ relates to the thermal diffusion from the multilayer to the substrate and ambient environment [40,41,42].

The control-pulse-fluence-dependent experimental data $\Delta E_{\mathrm{peak}}\left(\Delta t\right)/E_{\mathrm{peak}}$ can be well fitted using Equation (1), as plotted by the black curves in Figure 3a. Figure 3b presents the extracted values of the ultrafast demagnetization time constant $\tau_{m}$ under a positive magnetic field as a function of the control pulse fluence. It can be found that $\tau_{m}$ exhibits an independence on the pump fluence. The average demagnetization time is determined to be $\tau_{m}$ ≈ 0.42 ± 0.02 ps. This value is consistent with the previously reported ultrafast demagnetization time of rare-earth and transition-metal ferrimagnetic alloys. For example, in the Fe_74_Gd_26_ system, TR-XMCD yielded an Fe sublattice demagnetization time of ~0.47 ps, while the Gd sublattice achieved a demagnetization time of 0.90 ± 0.10 ps [43]. This indicates that the spin dynamics in Pt/Gd_19_(CoFe)_81_/Ta is dominated by 3d transition-metal electrons.

Table 1 presents three sets of time constant $\tau_{m}$, spin–lattice relaxation $\tau_{\mathrm{sl}}$ and heat diffusion $\tau_{d}$ values, obtained by various experimental techniques across different material systems. As shown in Table 1, $\tau_{m}$ is consistent with previously reported values, such as ~250 ± 30 fs for Fe/W [44], ~300 fs for FeGe [45] and 215–400 fs for Fe_60_Al_40_ [46].

Notably, the Pt/Gd19(CoFe)81/Ta system studied here has a relatively low Gd concentration, so its transient magnetic response is dominated by the CoFe transition-metal sublattice. Consequently, only a single demagnetization process is observed, unlike the two-step demagnetization typically reported for high-Gd-content systems [47]. For 3d ferromagnetic metals, ultrafast magnetization dynamics are generally attributed to electron–magnon scattering [48,49,50].

**Table 1:** Summary of time constants of UDM ($\tau_{m}$), and spin–lattice relaxation ($\tau_{\mathrm{sl}}$) and heat diffusion ($\tau_{d}$).

	Material	$\tau_{m}$ (fs)	$\tau_{sl}$ (ps)	$\tau_{d}$ (ps)	Methods	Refs.
3d ferromagnetic metals	Co	~260	/	/	TR-MOKE	[15]
	Ni	~160	/	/	TR-MOKE	[15]
	Fe	373 ± 20	0.38 ± 0.02–0.42 ± 0.01	/	OPTE	[31]
	Co	130–200	0.4–0.6	/	OPTE	[35]
	Fe/MgO(100)	50–75	0.8 ± 0.13	>100	TR-MOKE	[50]
	Fe/MgO	~200	/	/	TR-MOKE	[51]
	Fe/MgO(001)	<400	/	/	TR-MOKE	[52]
Magnetic alloys	FeGe(111)	~300	~4.8	/	TR-MOKE	[45]
	Fe_60_Al_40_	215 ± 50–400 ± 50	/	/	TR-MOKE	[46]
	L1_0_-FePt	~200	0.4–1.4	~100–260	TR-MOKE	[53]
	FeRh	275–325 (Fe) 275–350 (Rh)	/	/	TR-XUV-MOKE	[54]
	NiFe	~160 ± 30	/	~3–4.5	TR-MOKE	[55]
Ferromagnetic heterostructures	TbCo/Pt	120–280	0.3–0.7	/	OPTE	[35]
	Co/Pt	100–290	0.3–0.8	/	OPTE	[35]
	Fe/W(110)	250 ± 30	~2–9	/	TR-MOKE	[44]
	Fe/W(110)	~350–500	~4–7	/	TR-SPES	[45]
	Co_2_FeAl_0.5_Si_0.5_/Pt	~270	~0.8	~1300	TR-MOKE	[56]
	Co_2_FeAl_0.5_Si_0.5_/Ta	~280	~1.5	/	TR-MOKE	[56]
	Fe/Pt	280–300	/	/	Calculation	[57]
	Fe/Al	280–300	/	/	Calculation	[57]
	Fe/Au	350–400	/	/	Calculation	[57]
	Ta/Gd_0.22_(Co_0.8_Fe_0.2_)_0.78_/Al	~500	~1–3	/	TR-MOKE	[47]
	Ta/Gd_0.24_(Co_0.8_Fe_0.2_)_0.76_/Al	$\tau_{m1}:$ 1160 $\tau_{m2}:$ 15,000	>30	/	TR-MOKE	[47]
	Pt/Gd_19_(CoFe)_81_/Ta	420 ± 20	2.49 ± 0.11–3.28 ± 0.03	57.36 ± 11.28–164.96 ± 1.61	OPTE	This work

After demagnetization, the system enters the magnetic recovery process. Figure 3c,d show the pump fluence dependences of the fitted $\tau_{\mathrm{sl}}$ and $\tau_{d}$ values, respectively. Under +H, the $\tau_{\mathrm{sl}}$ of the Pt/Gd_19_(CoFe)_81_/Ta heterostructure increases from approximately 2.49 ± 0.11 ps to 3.28 ± 0.03 ps over the fluence range, being longer than that for lots of single ferromagnets, while being similar to that for FM/NM heterostructures, such as $\tau_{\mathrm{sl}}$ = 2–9 ps for Fe/W(110) multilayers probed by TR-MOKE [44]. The $\tau_{\mathrm{sl}}$ is related to the magnetic anisotropy. As the pump fluence increases, spin agitation is enhanced, resulting in a greater reduction in the transient magnetic anisotropy energy. The reduction in transient magnetic anisotropy is mainly responsible for the slower spin–lattice relaxation observed at higher pump fluence.

Additionally, the slow recovery time constant, $\tau_{d}$, increases from approximately 57.36 ± 11.28 ps to 164.96 ± 1.61 ps as the control pump fluence increases from 0.80 to 1.19 mJ/cm^2^. For comparison, values reported in previous works are $\tau_{d}$ > 100 ps for Fe/MgO(100) multilayers [50] and ~100–260 ps for L1_0_-FePt [53]. This indicates that the pump fluence used in this work is high enough to raise the temperature of the heterostructure. Therefore, the thermal energy transfer between the sample and the substrate generally takes a longer time under higher laser pump fluence in our experiment.

## 4. Conclusions

This work investigates the ultrafast spin dynamics of Pt/Gd_19_(CoFe)_81_/Ta heterostructures grown on SiO_2_ substrate using double-pump optical pump–terahertz emission spectroscopy. The experimental results clearly resolve a single-step demagnetization process with a characteristic time of approximately 0.42 ps, followed by two distinct magnetic recovery components occurring on different timescales. The extracted demagnetization time is consistent with the typical timescale of 3d transition-metal sublattices, indicating that the transient magnetic response is dominated by the CoFe sublattice. Our results demonstrate that OPTE provides a complemental approach for characterizing multi-timescale coupling processes in ferrimagnetic heterostructures on the femtosecond timescale, offering experimental insights for ultrafast spin manipulation [58] and THz device engineering [59,60].

## Data Availability

The original contributions presented in this study are included in the article. Further inquiries can be directed to the corresponding authors.
